# Epidemiology, Co-Infections, and Outcomes of Viral Pneumonia in Adults: An Observational Cohort Study

**DOI:** 10.1097/MD.0000000000002332

**Published:** 2015-12-18

**Authors:** Matthew P. Crotty, Shelby Meyers, Nicholas Hampton, Stephanie Bledsoe, David J. Ritchie, Richard S. Buller, Gregory A. Storch, Scott T. Micek, Marin H. Kollef

**Affiliations:** From the Pharmacy St. Louis College of Pharmacy (STLCOP) (MPC); STLCOP and Dept of Pharmacy, Barnes-Jewish Hospital (DJR); STLCOP (SM); STLCOP and Dept of Pharmacy, Barnes-Jewish Hospital (STM); Center for Clinical Excellence, BJC Healthcare (NH); Department of Pediatrics, Washington University School of Medicine (RSB, GAS); and Division of Pulmonary and Critical Care Medicine, Washington University School of Medicine (MHK).

## Abstract

Advanced technologies using polymerase-chain reaction have allowed for increased recognition of viral respiratory infections including pneumonia. Co-infections have been described for several respiratory viruses, especially with influenza. Outcomes of viral pneumonia, including cases with co-infections, have not been well described.

This was observational cohort study conducted to describe hospitalized patients with viral pneumonia including co-infections, clinical outcomes, and predictors of mortality. Patients admitted from March 2013 to November 2014 with a positive respiratory virus panel (RVP) and radiographic findings of pneumonia within 48 h of the index RVP were included. Co-respiratory infection (CRI) was defined as any organism identification from a respiratory specimen within 3 days of the index RVP. Predictors of in-hospital mortality on univariate analysis were evaluated in a multivariate model.

Of 284 patients with viral pneumonia, a majority (51.8%) were immunocompromised. A total of 84 patients (29.6%) were found to have a CRI with 48 (57.6%) having a bacterial CRI. Viral CRI with HSV, CMV, or both occurred in 28 patients (33.3%). Fungal (16.7%) and other CRIs (7.1%) were less common. Many patients required mechanical ventilation (54%) and vasopressor support (36%). Overall in-hospital mortality was high (23.2%) and readmissions were common with several patients re-hospitalized within 30 (21.1%) and 90 days (36.7%) of discharge. Predictors of in-hospital mortality on multivariate regression included severity of illness factors, stem-cell transplant, and identification of multiple respiratory viruses. In conclusion, hospital mortality is high among adult patients with viral pneumonia and patients with multiple respiratory viruses identified may be at a higher risk.

## INTRODUCTION

Viral pneumonia and lower respiratory tract infections are increasingly being recognized in adult patients including the critically ill.^1,2^ It appears that most viral lower respiratory tract infections are community-acquired and account for a significant etiology in mechanically ventilated patients with severe community-acquired pneumonia.^3,4^ Bacterial-viral co-infections are best described with influenza. The long history of bacterial infections occurring concurrently or shortly after influenza illness dates back to the 1918 influenza pandemic in which most of the fatal cases were found to be due to co-infection based on autopsy findings.^5^ More recently the 2009 H1N1 influenza pandemic was complicated by bacterial pneumonia in 4% to 33% of hospitalized or critically ill patients.^6–11^ Most commonly isolated co-infecting bacterial organisms with influenza are *Streptococcus pneumoniae*, *Staphylococcus aureus*, *S. pyogenes*, and *Haemophilus influenzae*. Influenza seasons are not equal as some are associated with lower mortality potentially related to differences in virulence factors or other unknown reasons.^12–15^

Bacterial co-infection is not limited to influenza and has been described with numerous other respiratory viruses, including respiratory syncytial virus (RSV), parainfluenza virus (PIV), rhinovirus, adenovirus, and human metapneumovirus (hMPV).^16–25^Advanced technologies have allowed for increased recognition of viral pathogens and diagnoses of viral respiratory infections including pneumonia.^26^ Several mechanisms by which viral respiratory infections may predispose patients to bacterial co-infections have been investigated including virus-induced alterations in epithelial cells, impaired immune response, and enhanced bacterial colonization.^27^ Utilizing new diagnostic technologies, it may be possible to better describe the clinical aspects of viral pneumonia and interactions with other infecting organisms. The purpose of this study was to describe hospitalized adult patients with viral pneumonia including possible co-infections and clinical outcomes.

## METHODS

### Subjects and Study Design

This was a single-center, observational cohort study of patients with a positive respiratory virus panel (RVP) at Barnes-Jewish Hospital (a 1300-bed urban academic medical center in St. Louis, MO) between 1 March 2013 and 7 November 2014. The study protocol was approved by the Barnes-Jewish Hospital, Washington University and St. Louis College of Pharmacy Institutional Review Boards. Adult patients (≥19 years of age) admitted to the hospital for >24 h were identified through a query of an internal database, which tracks respiratory viruses and evaluated for study inclusion. Patients were excluded if no virus was identified by RVP, rhinovirus or enterovirus was identified by nasopharyngeal (NP) swab only, or if a respiratory virus had been identified within the 90 days before the index RVP.

### Respiratory Virus Panel

The FilmArray^®^ respiratory panel assay (BioFire Diagnostics, Salt Lake City, UT) is a multiplex nucleic acid test capable of simultaneous qualitative detection and identification of multiple respiratory viral and bacterial nucleic acids. This panel became the primary diagnostic RVP used at BJH in March of 2013 and is capable of detecting 20 total respiratory pathogens (17 viral and 3 bacterial): *Bordetella pertussis, Chlamydophilapneumoniae, Mycoplasma pneumoniae,* Adenovirus, Coronavirus HKU1, Coronavirus NL63, Coronavirus 229E, Coronavirus OC43, Influenza A, Influenza A subtype H1, Influenza A subtype H3, Influenza A subtype 2009 H1, Influenza B, hMPV, PIV 1, PIV 2, PIV 3, PIV 4, RSV, and rhinovirus/enterovirus. The assay cannot reliably differentiate between human rhinovirus and enterovirus due to their genetic similarity. The FilmArray^®^ respiratory panel assay is FDA approved for NP swabs and additional sample types have been validated internally.

## DEFINITIONS

Viral pneumonia was defined as identification of a respiratory virus by RVP and a new or progressive radiographic infiltrate within 48 h. Respiratory co-infection (RCI) was defined as identification of a respiratory pathogen from a specimen obtained within 72 h of the index RVP. RCIs were stratified as bacterial, viral (HSV or CMV), fungal, or other RCI, which included *Mycobacteria* spp. and *Pneumocystis jiroveci. Candida* spp. isolated from respiratory specimens were excluded from RCI consideration. Immunocompromised status was defined as a diagnosis of HIV, active malignancy (stem cell transplant or receiving chemotherapy), solid organ transplant, or currently receiving immunosuppressive therapy (prednisone 20 mg/day for at least 30 days or equivalent). Determination of APACHE II scores was based on physical and laboratory findings on the day the index RVP was obtained. Charlson's comorbidity index was used as a summative score of underlying disease states.^28^ Electronic medical record (eMR) queries were used to acquire patient information where possible. Manual chart review was used to validate and supplement all eMR queries.

### Microbiologic Evaluation

Information regarding RVPs including time of collection/report, type of specimen, patient location at time of collection, and resulting findings were obtained from an internal database. Additionally, all available aerobic and anaerobic bacterial cultures were evaluated as available based on eMR query. Urine antigens for *Legionella*, direct-fluorescent antibody for *Pneumocystis jiroveci*, and *Clostridium difficile* toxin assay were also evaluated. In vitro susceptibilities were evaluated as reported per institutional practices.

### Endpoints

The primary endpoint evaluated was in-hospital mortality. Secondary endpoints included hospital length-of-stay (LOS), intensive care unit (ICU) admission, and readmission rates at 30, 90, and 180 days after the index hospitalization.

### Statistical Analysis

Descriptive statistics were used to describe the overall population of adult patients with viral pneumonia. Univariate analyses were then performed to compare survivors and nonsurvivors. Categorical variables were compared using chi square or Fisher's exact test as appropriate. Continuous variables were compared using the Mann–Whitney *U* test. All tests were 2-tailed and *P* values <0.05 were considered significant. Multivariable logistic regression analysis was then used to determine factors independently associated with in-hospital mortality. Variables with *P* values <0.20 on univariate analyses were entered into a multivariable model using a backward stepwise approach. All statistical analyses were performed using SPSS (version 22.0; Chicago, IL).

## RESULTS

### Study Population and Patient Characteristics

**FIGURE 1:**
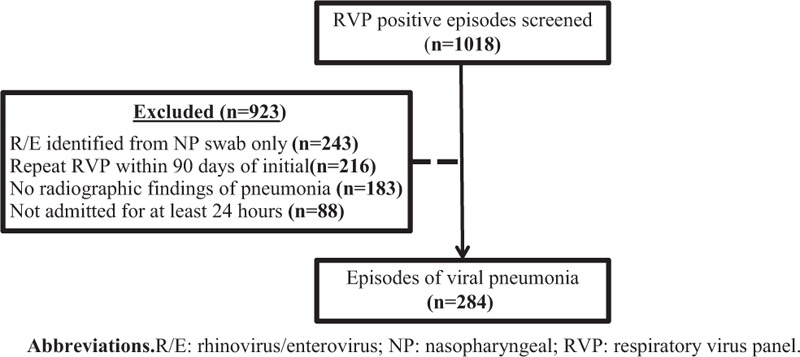
Identification of study population.

A total of 284 adult patients were identified as having viral pneumonia in this study (Fig. 1). Demographics and clinic characteristics are summarized in Table 1. There were slightly more women than men and most patients were Caucasian (66.9%). A majority of patients were immunocompromised (51.8%) with active malignancy (64.6%) the most common etiology followed by solid organ transplant (23.1%). Nearly all patients had at least 1 chronic comorbid condition (84.2%) with several having >1 (47.9%). Diabetes mellitus, chronic kidney disease, and chronic obstructive pulmonary disease were the most commonly identified comorbid conditions. Few patients had been hospitalized in the 90 days prior to the index admission (3.2%).

**TABLE 1:**
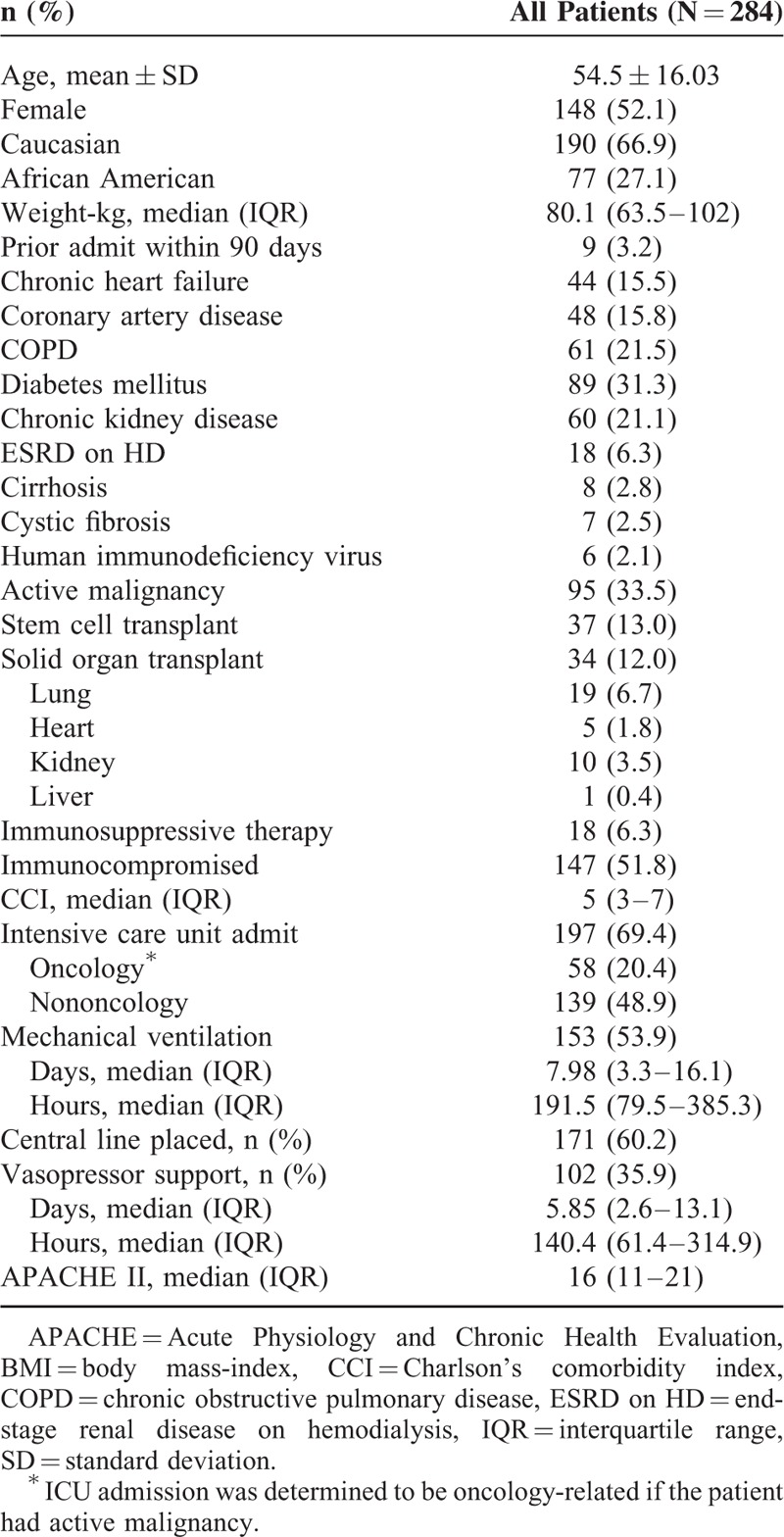
Demographic and Clinical Characteristics of Adult Patients With Viral Pneumonia

Most patients were critically ill (69.4%) with severity of illness also very high as illustrated by the median APACHE II score of 16. A majority of patients were mechanically ventilated (53.9%) and several required vasopressor support (35.9%).

### Virus Identification

**FIGURE 2:**
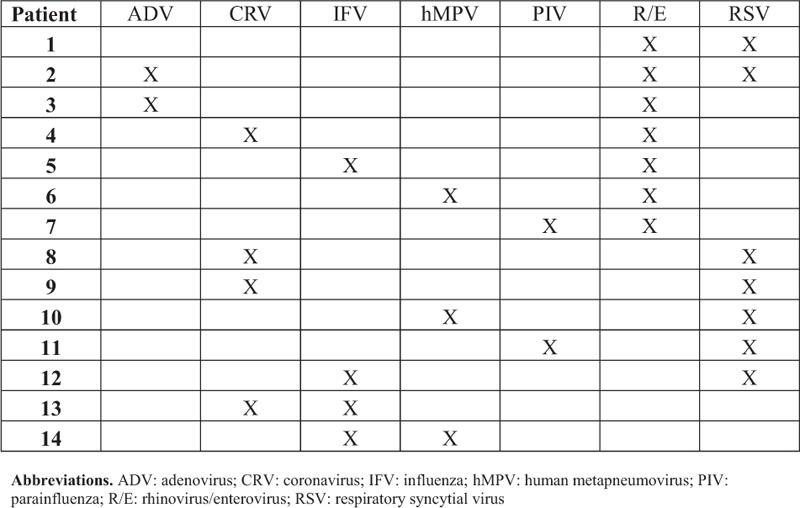
Description of patients with multiple respiratory viruses identified.

The most common viruses identified were influenza (24.3%), rhinovirus or enterovirus (23.6%), PIV (13%), and RSV (10.6%) (Table 2). Several (n = 14) patients had multiple viruses identified by RVP (Fig. 2). RSV (50%), rhinovirus/enterovirus (50%), influenza (28.6%) and coronavirus (28.6%) were most commonly identified among patients with multiple viruses identified by RVP. Most patients with multiple viruses were immunocompromised (10 of 14): 6 with active malignancy (4 with stem-cell transplants), 3 with lung transplants, and 1 patient with AIDS. Among the 73 patients with influenza, 56.2% (n = 41) received oseltamivir and 16.4% (n = 12) received investigational intravenous zanamivir. Inhaled ribavirin was administered to 21.6% (n = 8) of patients with RSV and 4.6% (n = 13) of patients overall. Other indications for inhaled ribavirin included hMPV, PIV, and rhinovirus/enterovirus.

**TABLE 2:**
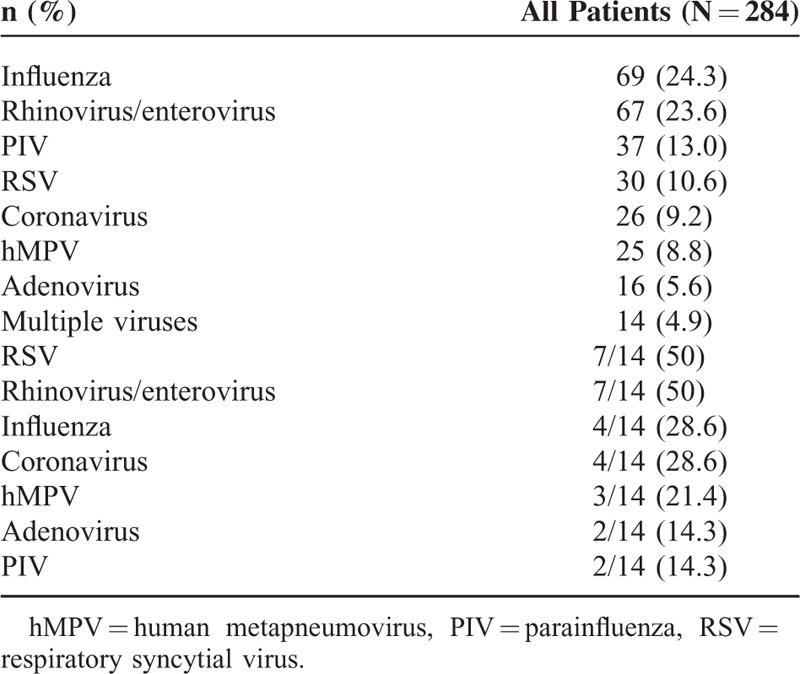
Viruses Identified in Patients With Viral Pneumonia

### Respiratory Co-infections

**FIGURE 3:**
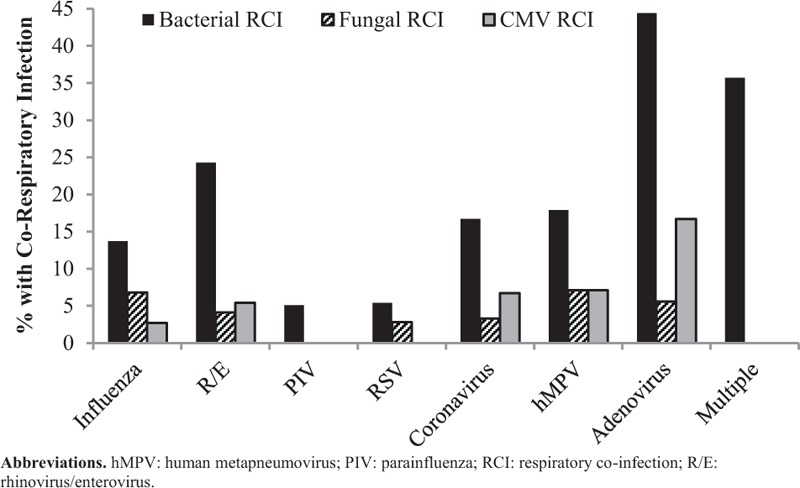
Respiratory co-infection rates according to virus identified.

A total of 84 (29.6%) of patients with viral pneumonia had an identified RCI with bacterial co-infection (57.1%) being most common (Table 3). Patients with adenovirus or multiple viruses identified by RVP were most likely to have a bacterial RCI (Fig. 3). Patients with viral pneumonia due to PIV, RSV, or influenza were least likely to have a bacterial RCI. Only 1.1% (n = 3) patients went on to have a subsequent bacterial RCI with all occurring 4 to 7 days after the index RVP report date. One-third of patients (n = 28) with an RCI had either HSV or CMV co-infection. Patients with adenovirus pneumonia were most likely to have CMV RCI. Rates of CMV RCI did not differ much among the other RVP virus types. A total of 14 patients (4.9%) had an identified fungal RCI, 50% (n = 7) were immunocompromised with 36% (n = 5) having active malignancy. *Aspergillus* sp. were the most common fungal organisms seen (50%; n = 7). Other fungi identified included *Arthrographis* sp., *Bipolaris* sp., *Paecilomyces* sp., *Scedosporium* sp., and *Dematiaceous fungi imperfecti*. Fungal RCI rates were similar among the different viruses identified by RVP. Few patients (1.8%; n = 5) had mycobacterial RCI and only 1 patient (0.4%) hadconcomitant *Pneumocystis jiroveci* pneumonia identified.

**TABLE 3:**
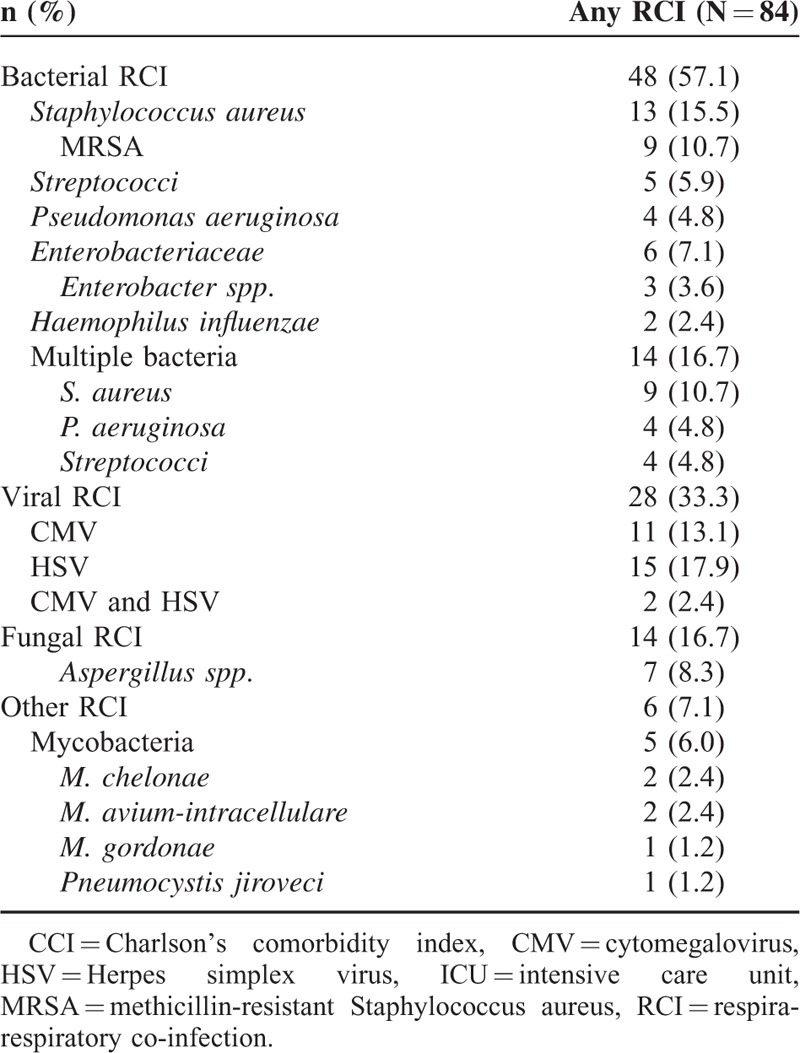
Respiratory Co-infections of Patients With Viral Pneumonia

### Clinical Outcomes

The multivariate model used to evaluate in-hospital mortality had an acceptable Hosmer–Lemeshow goodness-of fit test (*P* = 0.837) and fit the data well with an area under the receiver operating characteristic curve of 0.864 (Table 4). Variables identified as being associated with in-hospital mortality included primarily severity of illness factors: intensive care unit (ICU) admission, APACHE II score, and vasopressor requirement. Stem-cell transplant (aOR: 4.22; *P* = 0.004) and identification of multiple viruses by RVP (aOR: 4.87; *P* = 0.038) were also associated with mortality.

**TABLE 4:**
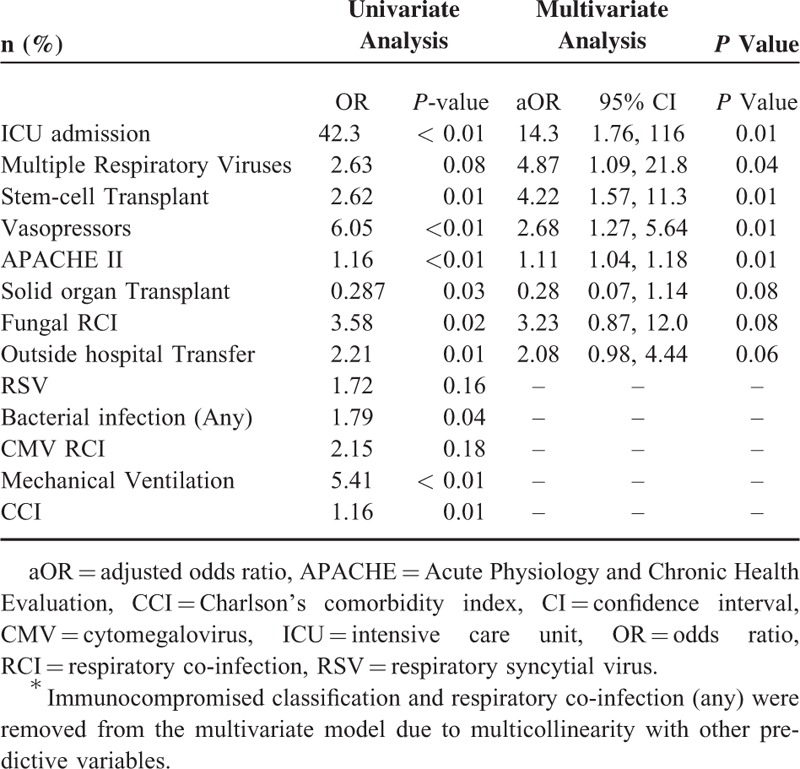
Predictors of In-Hospital Mortality by Univariate and Multivariate Logistic Regression^∗^

Overall in-hospital mortality was high (23.2%) with all but 1 patient being admitted to the ICU (98.5%). In-hospital mortality was higher among ICU patients with active malignancy compared to other ICU patients at 48.3% and 26.6%, respectively (*P* = 0.003). ICU length-of-stay was variable (median: 10.3 days; IQR: 4.4, 20.7) as was total hospital length-of-stay (median: 12 days; IQR: 5, 25.75). Readmissions were common with several patients re-hospitalized within 30 (21.1%) and 90-days (36.7%) of discharge.

## DISCUSSION

This study described 284 hospitalized adult patients with viral pneumonia and assessed factors associated with increased in-hospital mortality. Identification of multiple viruses by RVP, stem-cell transplant, ICU admission, APACHE II score, and requirement of vasopressors were all significantly associated with in-hospital mortality in a multivariable model. Several patients (29.6%; n = 84) had RCIs with most being bacterial (57.1%; n = 48). Patients with viral pneumonia due to adenovirus or multiple respiratory viruses were most likely to have a bacterial RCI.

Increasing recognition of viruses as pathogens in lower respiratory tract infections and pneumonia has altered the landscape of this illness. In our study, severity of illness characteristics were identified as being the primary factors associated with in-hospital mortality among patients with viral pneumonia. Interestingly, identification of multiple respiratory viruses was also associated with increased mortality. A potential explanation for this finding is that host characteristics enabling infection of multiple respiratory viruses may lead to worse outcomes. This finding may be a surrogate marker for immunocompromise or debilitation status of such patients and thus, their increased risk of death. However, it is also possible that co-infection with multiple viruses leads to a more pathogenic or detrimental respiratory process.

Bacterial co-infection was not found to be associated with in-hospital mortality in this study. Choi et al previously found no difference in 28-day mortality between ICU patients with severe bacterial or viral pneumonia at 25.5% and 26.5%, respectively.^30^ There was no pure bacterial pneumonia comparator group in our study and so no direct comparison in outcomes can be made between viral and bacterial pneumonia. Our results do suggest that clinical outcomes of viral pneumonia may not be substantially impacted by the presence of co-infecting bacteria. However, antibacterial therapy in this population was not thoroughly evaluated for appropriateness which would likely impact clinical outcomes of patients with a bacterial RCI.

Bacterial RCI occurred in 16.9% of patients with *S. aureus* and streptococci being the most commonly identified organisms. In a prospective study of mechanically ventilated patients with severe community-acquired pneumonia by Karhu et al, *S. pneumoniae* was the most frequently identified co-infecting bacterial pathogen (68.4%; n = 13) among patients with a mixed viral-bacterial pneumonia.^4^ Additionally, this study found more frequent bacterial co-infection with only 5 patients (11.1%) having a pure viral infection compared to 19 (42.2%) with mixed viral-bacterial infections. Our study was not limited to community-acquired infections which may account for this disparity; however, other studies have also reported lower rates of bacterial co-infection.^29,30^ Additionally, it is unlikely that a higher incidence of healthcare-related infections would explain the lower rates of bacterial co-infection observed.

Several limitations of our study should be recognized. First, the retrospective design did not allow for determination of the cause of mortality. Furthermore, antibacterial administration before respiratory culture obtainment was not evaluated and could have impacted rates of bacterial RCI seen. Second, patients transferred from an outside hospital may have had additional infectious processes identified by cultures taken outside of our hospital system. In such cases, co-infections may have been missed and falsely decreased the rates observed. Third, viral shedding may occur for several days to weeks after initial respiratory virus infection especially among immunocompromised patients. Our study classified viral pneumonia based on the index RVP report date and radiographic evidence of pneumonia. It is possible that for some patients the initial viral infection had occurred days before identification by RVP and that a concomitant process was detected on imaging. In this case, more bacterial RCIs would be classified as subsequent infections. Fourth, all cases in this study occurred in a 20-month period at a single institution. Viral epidemiology at this site and during this timeframe may not be representative of all seasons and locations. Finally, due to the constraints of the RVP used, we may have not identified all possible viral infections such as bocavirus.

Respiratory viruses continue to be frequently identified in hospitalized patients as new technologies push diagnostic abilities forward. This study describes hospitalized adult patients with viral pneumonia and factors associated with in-hospital mortality. Identification of multiple respiratory viruses and several factors describing severity of illness were associated with in-hospital mortality in a multivariate model. Recognition of multiple viruses as a poor prognostic indicator is of interest. With increased ability to recognize viral pathogens, this finding could simply be the result of improved diagnostics. However, it may also be indicative of host factors allowing for simultaneous infection by multiple respiratory viruses. In this study, only 16.9% of patients with viral pneumonia had a bacterial RCI. Use of antibacterials in this setting was not evaluated, but future research should focus on the role of empiric antibacterial use in patients with only viral infection identified. Additionally, procalcitonin has been shown to be useful in differentiating viral infections from bacterial and could prove useful in conjunction with identification of a respiratory virus in this population.^4,31,32^ In conclusion, this study adds to the growing body of literature regarding the epidemiology of viral pneumonia in adult patients and illustrates the high morbidity and mortality associated with viral pneumonia in adult patients. Additionally, this study suggests that identification of multiple infecting respiratory viruses is a negative prognostic indicator as illustrated by its association with in-hospital mortality. Continued advances in diagnostic technology have enabled more thorough evaluation of respiratory infections. Further investigation into the role viruses play in pneumonia may unclose more answers regarding patient risk factors for and outcomes of viral pneumonia.
